# From “Mental/Psychological Disorder” and/or “Individual Pattern of Adaptation” Toward “Developmental Trajectories During the Lifespan”

**DOI:** 10.3390/bs15050591

**Published:** 2025-04-28

**Authors:** Donatella R. Petretto, Alessandro Mura, Mattia Vacca, Gian Pietro Carrogu, Luca Gaviano, Riccardo Atzori, Carmelo Masala

**Affiliations:** Department of Education, Philosophy, Psychology State University of Cagliari, 09127 Cagliari, Italy; a.mura111@studenti.unica.it (A.M.); mattiavacca67@gmail.com (M.V.); gpcarrogu@gmail.com (G.P.C.); luca.gvn@gmail.com (L.G.); riccardoatzori@hotmail.it (R.A.); masacarmelo@gmail.com (C.M.)

**Keywords:** mental disorder, psychiatric disorder, psychological disorder, individual pattern of adaptation, pattern of maladaptation, protective factors, risk factors, developmental trajectories

## Abstract

A general and still open question in clinical psychology is the crucial one: “What is a psychological/mental disorder?”. In the second half of the 20th century and now, the question has been addressed along two different parallel paths/approaches: one path aimed to classify those disorders, also thanks to the development of the international classification manuals (DSM and ICD); another path aimed to describe in what ways those “disorders” manifest themselves in the life of each individual. While the first path used explicitly the word “disorder”, the second one mainly preferred to use the words “pattern of adaptation/maladaptation”. In this brief perspective paper, we will discuss both paths and approaches, according to the perspective of clinical psychology and as a result of a narrative review. We discuss some differences between the two paths, some points of contact between them, and some critical issues. Moreover, we will briefly discuss a third integrative path that may integrate the first two. We then discussed if the third integrative path may increase the strength of both previous ones and overcome their limits, with the aim to support individual patterns of adjustment, prevent “mental/psychological disorder” and empower individuals in coping with adverse life events during all the phases of lifespan.

## 1. Introduction

A general and still open question in clinical psychology is a crucial one: “What is a psychological/mental disorder?” Since ancient times, clinicians and researchers have tried to answer this question, with different approaches and various results.

From the second half of the 20th century to the present, this question has been addressed along two main different but parallel paths: one aims to describe and classify psychological/mental disorders, aided by the development of international classification manuals (DSM and ICD) (APA, 2022; World Health Organization [WHO], 2020); the other path aims to describe the ways in which these “disorders” manifest themselves in the life of each individual (see Achenbach, 1974; Cicchetti, 2006; Hyde et al., 2024; Masten, 2024; Shaw et al., 2024).

While the first path explicitly uses the word “disorder”, the second one mainly favors the phrase “pattern of adaptation”. In the first path, clinicians use certain words to better describe disorders, namely, “psychological”, “psychic”, “mental”, and “psychiatric”, with the aim of better defining the “gradient of severity” of the disorder itself. In the second path, clinicians specify whether the “pattern of adaptation” is “adaptive” or “maladaptive”: the same pattern of adaptation can be “adaptive” or “maladadaptive”, and its effects change during various phases of life (Sroufe & Rutter, 1984; Cicchetti, 2006).

Both paths were initiated in the same year during the 1970s, soon after a great debate on the idea of “mental disorder”, on its classification, on the need to classify disorders (after the renewed interest in the so-called Kraepelinian and neo-Kraepelinian approaches) and based on the need to find the correct balance among psychiatric, psychological, and psychoanalytic frameworks for psychological disorders and distress (Mack et al., 1994; Decker, 2007; Brückner, 2023).

In this brief perspective article, we discuss both paths and approaches and briefly examine a third path that may integrate the first two. This third path is based on the construct of “developmental trajectories” and on the continuity/discontinuity between different patterns of adaptation during different phases of the lifespan and/or between different diagnoses of mental disorders during the lifespan. This third path may act as a base to describe and then support “individual patterns of adjustment” to prevent the development of a “mental/psychological disorder” or crisis when individuals have to cope with adverse life events. In the following, we then explore whether this third integrative path may overcome the limits of the previous ones and whether it may be used to organize prevention interventions and to organize interventions aimed at empowering individuals to cope with adverse life events during all phases of their lives.

## 2. First Path: Definitions and Classifications of “Mental Disorders”

The first path aims to describe and classify psychological/mental disorders, aided by the development of international classification manuals (DSM and ICD) (APA, 2022; World Health Organization [WHO], 2020). Since its first inception, attention in psychopathology has been focused on the need to describe signs, symptoms, and syndromes and classify group(s) of disorders that share the same signs and symptoms according to a descriptive approach. Starting from Kraepelin’s proposal and the later need to have diagnostic criteria for statistical and treatment purposes, some scholars tried to create shared diagnostic criteria (for example, the “Research Diagnostic Criteria”) and a shared vision of “disorder” related to psychological and mental fields (Spitzer & Endicott, 1978; Spitzer et al., 1978; Williams, 1982). Over time, they integrated various previous and concurrent approaches and theoretical positions (Mack et al., 1994; Decker, 2007; Brückner, 2023).

Together with the development of this first path, an open debate started—and is still ongoing—regarding the definition of “disorder” itself. Later, some definitions were developed and discussed but without a shared outcome, starting from Spitzer and Endicott’s definition (Spitzer & Endicott, 1978) and Wakefield’s definition (Wakefield, 1992a, 1992b; Petretto et al., 2025; Vacca et al., submitted). Moreover, in international statistical manuals, some definitions of mental disorders were proposed; for example, the DSM’s definitions and some other tentative definitions reinvigorated the debate, with a focus on specific topics/issues to be considered in the definition of disorder (etiology, clinical manifestations in various domains of functioning, and consequences for individual lives and adjustment) (Petretto et al., 2025; Vacca et al., submitted). In this first path, scholars chose to use the terminology “mental disorder” instead of “psychological disorder”, more for the sake of agreement than as a clear theoretical choice: the choice of the word “mental” was a compromise between a previous proposal (“psychiatric disorder”) and the need to satisfy the request of the American Psychological Association (“psychological disorder”). Undoubtedly, in the past, there was a need to find a correct balance between the need to declare the role of medicine in relation to the so-called “mental disorder” and the role of psychology, and the choice of “mental disorder” was the direct consequence of this need (Follette & Houts, 1996).

With reference to phases of life, the main focus was on the “clinical onset” of a disorder in a specific moment of the individual’s life, specifically the moment when the behavioral features of the disorders become “clinically” significant. Since the inception of the first path, clinicians have been very interested in specific moments of the individual’s life; that is, when something changes and when the individual needs support. In these moments, individuals may manifest clinically relevant features (in the cognitive, affective, and behavioral domains) and certain known signs and symptoms, sometimes due to a specific crisis related to an adverse life event and/or a subjective negative life event. Disorders were first distinguished according to the phase of life (infancy disorders, adult disorders, personality disorders, and so on) (as in DSM-IV (APA, 1994)), and only later does any reference to a lifelong approach appear in this path (as in DSM-5 (APA, 2013)). The first path has been well represented over the years, and it is still represented in more recent international diagnostic manuals (DSM-5-tr) (APA, 2022). A large amount of work has been conducted on the study of specific disorders, their clinical manifestations, etiology, consequences for individual lives, and intervention; however, more work is required. Most specifically, more work is required on the following fields: the need to find a correct balance between the categorical approach and the dimensional approach in the diagnosis of mental disorders, the need to distinguish between pathognomonic signs and symptoms of specific disorders, and the use of polythetic diagnostic criteria (aiming to describe the significant clinical heterogeneity seen in some disorders) (Petretto et al., 2025).

## 3. Second Path: “Individual Pattern of Adjustment”

In the second path, the focus is not on specific moment(s) of the individual’s life; rather, attention is focused on the lifespan and directed toward the need to describe development, continuity, and discontinuity in the “individual pattern of adjustment” in different phases of life (Sroufe & Rutter, 1984; Cicchetti, 2006; Hyde et al., 2024; Masten, 2024; Shaw et al., 2024). Similarly to the first path, the focus may first be placed on a specific (critical) moment(s) or transition(s) of the individual’s life, mainly the moment when the “individual pattern of adjustment” may not be relied on to completely overcome difficulties and cope with “specific life events”. Due to such difficulties, individuals may manifest distress and/or clinically significant behavioral features. At such moments, the individual may require a clinical consultation.

The peculiar feature of this second path is that, starting from this “specific critical moment”, the focus shifts to all the other phases of life, aiming to describe continuity and discontinuity in the “individual pattern of adjustment” (Sroufe & Rutter, 1984; Cicchetti, 2006). From this perspective, even if this second path starts to analyze the pattern of adjustment in a specific phase/moment of individual life, it also aims to analyze all the phases of a lifespan.

The second path was also developed in the 1970s. In the first years of this path, authors aimed to describe the preventability of adult disorders starting from infant and childhood disorders; only later did they start to describe the patterns of adjustment in various moments of an individual’s life and/or connections between these moments (Sroufe & Rutter, 1984; Cicchetti, 2006). In the initial studies, these moments were mainly related to infancy and adolescence; only later and in recent times have authors explored the whole lifespan and aimed to reconstruct continuity and discontinuity among various phases of life (Sroufe & Rutter, 1984; Cicchetti, 2006).

Regarding this second path, together with the development of this approach, there has been a great debate, which is still ongoing, on the related theoretical frameworks. In this scenario, “Developmental Psychopathology” emerged in the 1970s (Achenbach, 1974): it integrated various previous and concurrent approaches and theoretical positions. According to this approach, “psychopathology” is a complex process that unfolds during a lifespan. Protective and risk factors may have a role in this complex process and may influence and modify individual psychopathological trajectories. In recent years, significant work has been conducted on the definition of a complex causal model that considers individual features (genetic and constitutional level, phenotypic level, and behavioral level) and the interaction with environmental protective and risk factors (A. Venta et al., 2021). The role of developmental timing effects and the relationship between various phases of life are receiving the attention of scholars and clinicians. Some other issues are being discussed, with reference to the need to integrate various other theoretical frameworks (for example, the neuroscientific one) (Masten, 2006, 2024; Hyde et al., 2024; A. C. Venta & Marshall, 2022).

Also in this second path, a large amount of work has been conducted; however, more work is required. Most specifically, more work is required on the following fields: the description of continuity/discontinuity in the individual pattern of adjustment in all the phases of lifespan; the need to study and describe dimensional bases of continuity and discontinuity; and the need to study and describe in a deeper way the complex causal model that considers individual features (genetic and constitutional level, phenotypic level, and behavioral level) and the interaction with environmental protective and risk factors.

## 4. Points of Contact and Differences Between the Two Paths/Approaches

Even if the two paths are sometimes seen as different, there are various points of contact between them. The first is the “specific moment” of consultation between individuals and professionals. In both paths, the “primum movens” of the consultation always occurs at a specific moment of life where the overall ability to cope with negative (or subjectively negative) situations is overloaded. However, while clinicians refer to this moment in the first path as one involving the use of clinical and diagnostic labels, in the second path, clinicians try to understand the peculiarity of this moment; for example, if there is a specific transition and/or adverse life event and/or subjective negative life event. Moreover, in the second path, clinicians try to find specific individual and contextual protective and resilient factors to support individuals in overcoming the situation; they also try to prevent and/or eliminate specific individual and contextual risk factors.

Another point of contact is the relationship between the onset of the disorder and a specific critical phase of life, which in the first path is described using the diathesis–stress hypothesis and in the second path is described using the concept of maladjustment pattern. In the first path, the negative interaction between individual vulnerability (mainly constitutional and genetic ones) and the demand placed on an individual by specific life events is considered, while in the second path, the negative interaction between individual patterns of adjustment and the balance between protective and risk factors is considered. In a similar way between the two paths, the complex interaction between an individual and life events (negative ones or subjectively negative ones) is sometimes considered a “trigger” for the onset of clinically relevant situations.

There are also various differences, but one of them could be considered the most important. The focus of attention of the first path is on a specific moment of life (one or more than one) and the use of clinical and diagnostic labels for this specific moment of life. From this regard, the use of a clinical diagnostic label could be similar to “take a picture/photo”, but while it is the photo taken in a specific moment of life and it represents the “clinical situation” only at a specific moment of life, occasionally there is a risk that it may become a “photo” used to represent other specific moments as well and/or the entire life, starting from these specific moments. In this regard also, the risk of stigmatization is very high. As a general trend, in the first approach, clinicians rarely address all the phases of the lifespan, and they are generally specialized in specific phases of life (infancy, adolescence, adulthood, or elderhood). In the second path, authors do not consider the “pattern of adjustment” only in the specific critical moments, and they do not consider only the single “photo”, as in the first path, but rather they are interested in various phases of the lifespan, and they look to the “movie of the life”, which is composed of various moments of the individual’s life that are related by some sort of continuity between the pattern of adjustment used by the individual: some of these moments may be clinically relevant, while others may not; they may be sub-clinically relevant (or not clinically relevant at all, if the pattern of adjustment helps the individual to cope with the “subjective stressful situation”). Specifically, clinicians of the second path focus on the lifespan (the “movie”), and then they “zoom” in on specific critical moments during the lifespan where “clinical manifestations” appear and when the individual requires clinical consultations and/or support. They are also interested in other moments during a lifespan when the individual uses a similar “pattern of adjustment” with a positive result.

## 5. Third Path: Developmental Trajectories During the Lifespan Help Prevent “Clinical Disorders”, Support a “Pattern of Adjustment”, and Cope with “Clinical Disorders”

After about 50 years of development of the two parallel paths described in this paper, a third integrative path is now needed, which may consider the strengths of each path, which may try to overcome their limits. This third path is needed to study and to project how to support individuals in preventing mental disorders and critical phases in individual life and (if any) aiming to cope with those critical phases and/or disorders. A lifespan approach in psychopathology based on developmental trajectories is needed, focused on continuity and discontinuity between specific patterns of adjustment (and/or maladjustment) and between modalities and traits used to cope with situations and to interact with oneself, other people, and situations. The terminology “developmental trajectory” in psychopathology refers to the specific trajectory in an individual’s life and to the general construct of “developmental trajectory” as a probabilistic way to study psychopathology in human life and during lifespan. The study of those patterns of continuity/discontinuity between different phases of life, starting both from critical phases and zooming in and out on previous and following phases of life, might offer a new way to think about an individual’s life and its relationship between disorders, distress, and crisis, and with adjustment, functioning and coping abilities as well. The study of those patterns of continuity/discontinuity between each diagnosis of “mental disorders” (if any) between different phases of life, which again is able to zoom in and out on previous and following phases of life, might offer a new way to think about an individual’s life and its relationship with patterns of adjustment and maladjustment.

Taking together continuity and discontinuity with the interaction with protective and risk factors within a complex multilevel probabilistic causal model, characterized by interdependence among the different levels (individual, social, and environmental ones) and by mutually reinforcing effects operating over time, the study of the individual paths during the phases of lifespan will help to describe the so-called “developmental trajectory”.

The study of those “developmental trajectories” between all the phases of the life cycle, rather than a focus on “specific critical steps” of an individual’s life, might offer a new way to think about how to support individual patterns of adjustment, as well as to prevent crises, distress, disorders, and any critical steps, and to empower individuals in coping with “adverse life events” during all the phases of the lifespan. The same might offer a way to think about the etiology of disorders and/or critical phases of life within the just-mentioned complex multilevel probabilistic causal model (Petretto et al., 2025). Moreover, this third path may be useful in addressing the question, “How can clinicians help people cope with psychological/mental disorders?” We also need a new way to think about prevention and intervention based on the role of individual and contextual risk and protective factors in a probabilistic, complex, and multilevel causal model of “disorders” and/or a “maladaptive pattern of adjustment”, which considers individual features (genetic, constitutional, and behavioral ones) and their interaction with individual and contextual risk and protective factors.

## 6. Future Research and Perspectives

Future research, together with consolidated clinical data and clinical experiences, may help to develop the third path, a lifespan approach in psychopathology, focused on developmental trajectories among all phases of life, along two main different but interrelated directions: the development of a general framework for understanding both continuity and discontinuity among the psychopathological features/patterns of adjustment throughout one’s life and the development of an overall approach for intervention.

Regarding the general framework, research and clinical data are needed to overcome some issues in the first and second paths and to increase knowledge on the developmental trajectories of patterns of adjustment and clinical disorders (if any) during all phases of life. Firstly, a general framework is needed to achieve the following:

- Increasing clinical and research data and knowledge on continuity and discontinuity for the dimensional features and personality traits underlying the “pattern of adjustment” during all phases of life;
- Increasing clinical and research data and knowledge on developmental trajectories (continuity and discontinuity for dimensional features and personality features and traits underlying “pattern of adjustment” and clinical disorders—if any—during all phases of life), according to a probabilistic complex and multilevel causal model; emerging technologies may have a role in those fields;
- Increasing clinical and research data and knowledge on the dimensional aspects of mental disorders and on the pathognomonic signs and symptoms of specific disorders (or groups of disorders) to promote more valid diagnostic criteria; emerging technologies may have a role in those fields;
- Discussing (and, if necessary, overcoming) the polythetic approach used in the current diagnostic criteria to promote more valid diagnostic criteria and prevent artificial comorbidity related to this approach;
- Increasing clinical and research data and knowledge on the interaction between individual features (genetic, constitutional, and behavioral ones) and individual and contextual risk and protective factors, according to the approach of developmental trajectories and during all phases of life. Emerging technologies may have a role in those fields.

Regarding the overall approach for intervention and the ability to take into account all the previously discussed aspects, the third path may offer a new way to think about prevention and intervention based on the role of individual and contextual risk and protective factors in a probabilistic, complex and multilevel causal model of “disorders” and/or a “maladaptive pattern of adjustment”, which considers individual features (genetic, constitutional, and behavioral ones) and their interaction with individual and contextual risk and protective factors.

Regarding prevention, researchers and clinicians should cooperate with communities, social workers, and self-advocacy associations to support people in increasing their resilience and promoting their ability to cope with negative life events/transitions, increasing protective factors and limiting the effects of individuals’ and societal risk and vulnerability factors. Regarding intervention, researchers and clinicians should collaborate with communities, social workers, and self-advocacy associations to help people increase their resilience and promote their ability to cope with clinical disorders, increasing protective factors and limiting the effects of individual and societal risk and vulnerability factors.

With these aims in mind, contributions are also needed from scientific societies, stakeholders, and health policymakers to support researchers and clinicians in developing the general framework and in planning and implementing the overall integrated path for prevention and intervention. Specific training programs for professionals in relevant fields are also needed.

## Data Availability

Not applicable.
